# Direct hydrogen production from dilute-acid pretreated sugarcane bagasse hydrolysate using the newly isolated *Thermoanaerobacterium thermosaccharolyticum* MJ1

**DOI:** 10.1186/s12934-017-0692-y

**Published:** 2017-05-03

**Authors:** Bin-Bin Hu, Ming-Jun Zhu

**Affiliations:** 1 School of Bioscience and Bioengineering, South China University of Technology, Guangzhou Higher Education Mega Center, Panyu, Guangzhou, 510006 People’s Republic of China; 2 School of Life and Geographical Sciences, Kashi University, 29 Xueyuan Road, Kashi, 844006 Xinjiang Uygur Autonomous Region People’s Republic of China

**Keywords:** *Thermoanaerobacterium thermosaccharolyticum* MJ1, Sugarcane bagasse, Dilute-acid pretreated sugarcane bagasse hydrolysate, Inhibitor tolerance, Hydrogen production

## Abstract

**Background:**

Energy shortage and environmental pollution are two severe global problems, and biological hydrogen production from lignocellulose shows great potential as a promising alternative biofuel to replace the fossil fuels. Currently, most studies on hydrogen production from lignocellulose concentrate on cellulolytic microbe, pretreatment method, process optimization and development of new raw materials. Due to no effective approaches to relieve the inhibiting effect of inhibitors, the acid pretreated lignocellulose hydrolysate was directly discarded and caused environmental problems, suggesting that isolation of inhibitor-tolerant strains may facilitate the utilization of acid pretreated lignocellulose hydrolysate.

**Results:**

Thermophilic bacteria for producing hydrogen from various kinds of sugars were screened, and the new strain named MJ1 was isolated from paper sludge, with 99% identity to *Thermoanaerobacterium thermosaccharolyticum* by 16S rRNA gene analysis. The hydrogen yields of 11.18, 4.25 and 2.15 mol-H_2_/mol sugar can be reached at an initial concentration of 5 g/L cellobiose, glucose and xylose, respectively. The main metabolites were acetate and butyrate. More important, MJ1 had an excellent tolerance to inhibitors of dilute-acid (1%, g/v) pretreated sugarcane bagasse hydrolysate (DAPSBH) and could efficiently utilize DAPSBH for hydrogen production without detoxication, with a production higher than that of pure sugars. The hydrogen could be quickly produced with the maximum hydrogen production reached at 24 h. The hydrogen production reached 39.64, 105.42, 111.75 and 110.44 mM at 20, 40, 60 and 80% of DAPSBH, respectively. Supplementation of CaCO_3_ enhanced the hydrogen production by 21.32% versus the control.

**Conclusions:**

These results demonstrate that MJ1 could directly utilize DAPSBH for biohydrogen production without detoxication and can serve as an excellent candidate for industrialization of hydrogen production from DAPSBH. The results also suggest that isolating unique strains from a particular environment offers an ideal way to conquer the related problems.

**Electronic supplementary material:**

The online version of this article (doi:10.1186/s12934-017-0692-y) contains supplementary material, which is available to authorized users.

## Background

High-quality modern life demands energy to sustain, and the importance of energy resources becomes apparent. However, with social progress, people have higher standards for the high-quality life, especially for the environment. Energy shortage and environmental pollution are two severe global problems [1]. Up to now, fossil fuels are still the main energy resources in the world and it is necessary to develop environmentally friendly and renewable energy resources.

Hydrogen is one of the most promising sustainable energies to replace the fossil fuels due to its high calorific value, environmental friendliness and efficient conversion to usable power [2]. When hydrogen is burnt, only water and energy are produced. A project has been started with the ultimate goal of promoting the transition into hydrogen in Taiwan [3]. Currently, the dominant technology for direct hydrogen production is gasification of heavy hydrocarbons, steam methane reforming, coal gasification, nuclear electrolysis, renewable electrolysis, power-grid electrolysis and pyrolysis [4]. However, these H_2_ production methods rely on high energy consumption, especially fossil fuels.

Bio-hydrogen production is an ideal technology for producing green hydrogen fuel, and bio-hydrogen has become a priority for most researchers and organizations. Dark fermentation of organic materials by bacteria presents a promising route of bio-hydrogen production, due to its environmental friendliness and high production [4, 5]. Low value feedstock and high capacity microorganisms are the two prime principles to reduce the cost of bio-hydrogen production [6]. Lignocellulosic feedstock is the most abundant and cheap resource in nature. Today, most of the lignocellulose residuals are directly burnt without effective utilization, which also causes serious environmental pollution [7]. The hydrolysate of lignocellulose is mainly composed of glucose and xylose that can be utilized for biofuel production by microorganisms.

The bio-hydrogen producing microorganisms which can utilize both glucose and xylose are considered as the promising candidate for industrialization. Among all the bio-hydrogen producing microbes, mesophilic bacteria have been studied extensively [8]. *Thermoanaerobacterium thermosaccharolyticum* (*T. thermosaccharolyticum*) is a well-known strain for bio-hydrogen production due to its ability to utilize various kinds of carbon sources including glucose, xylose, xylan, sucrose, cornstalk, Avicel, wheat straw, palm oil mill effluent, decanter cake and acid pretreated hydrolysate of corn stalk [8–13]. Dilute-acid hydrolysis is one of the pretreatment methods when using lignocellulose as the substrate. The acid hydrolysate usually contains not only soluble sugars but also significant quantities of inhibitors, such as acetate, phenol, and furan compounds that can adversely affect the microbial metabolism [14]. The acid hydrolysate can be detoxified by removal of inhibitors, but the treatment increases the production cost, thus the acid hydrolysate is usually discarded [13]. So far, some studies have investigated hydrogen production from non-detoxified hydrolysate by mixed consortia [15–18]. However, effective conversion of non-detoxified hydrolysate needs to be further studied.

The acid hydrolysate contains reducing sugars, and discarding it is not only a waste of resources, but also results in a pollution problem. The aim of the present study was to isolate, identify and characterize a thermophilic fermentative bacterium from paper sludge, which can make full use of the acid hydrolysate to produce valuable metabolites. The mechanism underlying the higher bio-hydrogen production from acid hydrolysate than pure reducing sugars was also investigated.

## Methods

### Isolation of the bacterial strain

Paper sludge was obtained from Zhongshun Paper Mill using commercial wood pulp with a kraft pulping process (Guangdong, China). To isolate the bacterial strain, 1 g paper sludge was mixed with 10 mL of sterile water and the supernatant was transferred to the modified MB medium under N_2_ atmosphere [19]. After 3 days of cultivation (55 °C, 150 rpm), the resultant culture broth was transferred at 10% (v/v) to fresh MB medium and cultured for another 3 days. When this enrichment process was repeated five times in the same manner, tenfold serial dilutions were mixed with the solid MB medium (2% agar, w/v) and rotated on the wall of the anaerobic tubes under N_2_ atmosphere. Subsequently, the tubes were incubated at 55 °C until the appearance of single bacterial colonies, followed by transferring the colonies to fresh MB liquid medium under N_2_ atmosphere by inoculation loop. The isolation procedure was repeated at least five times to ensure the purity of the isolated colonies. The isolated strain was stored in Guangdong Microbial Culture Center (GDMCC No. 60096).

### Strain identification

Genomic DNA was extracted using a Bacterial DNA Kit (Omega, USA) according to the manufacturer’s instructions. The extracted DNA was used as the template for PCR amplification of the 16S rRNA gene with a pair of universal primers: 16S-F (AGAGTTTGATCCTGGCTCAG) and 16S-R (ACGGTTACCTTGTTACGACTT). The PCR products were purified using a Gel and PCR Clean-up Kit (Omega, USA) and cloned into vector pMD18-T using the pMD18-T vector system I kit according to the manufacturer’s instructions (Takara, Dalian, China). The 16S rDNA was sequenced (Sangon Biotechnologies Co. Ltd., Shanghai, China) and aligned manually using the BLAST algorithm with all the nucleotide sequences deposited in the NCBI nucleotide database. Alignment was carried out using Clustal X [20]. The phylogenetic dendrogram was reconstructed using the MEGA program with the neighbor-joining algorithm and bootstrap analysis of 1000 replicates [21].

### Batch test of isolated strain with different reducing sugars

The effects of reducing sugar types on growth and hydrogen production were investigated using batch fermentations. The reducing sugars used were glucose, xylose, cellobiose and sucrose with a concentration of 5 g/L, initial pH of 7.00. All bottles were cultivated in a rotary shaker at 55 °C and 150 rpm. The growth curve determination was conducted in 10 mL penicillin bottles containing 4.5 mL of sterile MB medium and 10% inoculum. The OD_600_ value was measured at different intervals. All fermentation batches were conducted in 100 mL serum bottles with a working volume of 50 mL. The fermentation broth consisted of 45 mL of sterile MB medium and 10% inoculum. After 48 h incubation, the cell density, pH, residual carbon substrate concentration, hydrogen, and metabolic products in the broth were determined. For the hydrogen production from xylose, various xylose concentrations (2.5, 5.0, 7.5, 10 g/L) were adopted. All treatments were carried out in triplicate.

### Batch test of isolated strain with DAPSBH

Sugarcane bagasse (SCB) was obtained from Guangzhou Sugarcane Industry Research Institute (Guangzhou, China). The DAPSBH was obtained from the dilute acid pretreatment process of SCB. The raw SCB was soaked in sulfuric acid solution (1%, g/v) with a solid to liquid ratio of 1:10 (g dry weight to mL) at 121 °C for 30 min. After that, the DAPSBH was separated by vacuum filtration. The final DAPSBH was analyzed for soluble sugars (xylose and glucose) and by-products (formic acid, acetic acid and lactic acid). For the fermentability of DAPSBH, the DAPSBH was diluted by distilled water supplemented with the nutrients of MB medium (20, 40, 60 and 80%, v/v) except carbon source. All experiments were carried out in 100 mL serum bottles with a working volume of 50 mL composed of 45 mL DAPSBH medium and 5 mL seed liquid of *T. thermosaccharolyticum*. Under the same culture conditions as described above, the cell density, pH, residual carbon substrate concentration, hydrogen, and metabolic products in the broth were determined. To optimize the conditions (yeast extract concentration, inoculation ratio) for hydrogen production, 60% DAPSBH was adopted. The time course of hydrogen production, metabolite production and substrate consumption were determined by destructive sampling. All treatments were carried out in triplicate.

### Batch test of isolated strain with mixed sugars

To investigate the mechanism of higher hydrogen production when using DAPSBH as carbon source, the composition of reducing sugars in DAPSBH was simulated by adjusting the concentration of reducing sugars to ensure that the concentrations of glucose and xylose were equal to those in the DAPSBH. The simulation experiment (7.2 g/L xylose and 1 g/L glucose) was compared with 60% DAPSBH and xylose (8.2 g/L).

### Batch test of isolated strain with different pH, CaCO_3_ and buffer systems

In order to investigate the effect of pH on hydrogen production, the initial pH of fermentation medium was adjusted to 6.0, 6.5, 7.0, 7.5 and 8.0. To investigate the effect of CaCO_3_ on the hydrogen production, two concentrations of CaCO_3_ (20 and 40 mM) were employed in DAPSBH medium. After fermentation,the hydrogen and metabolites were determined. All treatments were carried out in triplicate. For buffer systems, phosphate buffer (PB) was used at three pH values (6.0, 6.5 and 7.0) and four concentrations (0, 0.1, 0.2 and 0.3 M). At the end of fermentation, the hydrogen and metabolites were determined. All treatments were carried out in triplicate.

### Analytical methods

Cell density in the liquid medium was monitored by measuring turbidity at 600 nm (GENESYS™ 10S, Thermo Fisher, United States). The micrograph of isolated strain was taken using an Atomic Force Microscope (AFM) at 15,000×.

Hydrogen was measured with a gas chromatograph (Fuli 9790, Fuli, China) equipped with a thermal conductivity detector (TCD) and a flame ionization detector (FID) through a TDX-01 column and an AE electric insulating oil analysis column using the method as described by Li et al. [22]. The column temperature was isothermally set at 60 °C. The carrier gas was N_2_ (35 mL/min) and 1 mL sample gas was used for detection. Hydrogen production quantity was deduced by the molar ratio of H_2_/N_2_.

The concentrations of soluble sugars, organic acid and ethanol in filtered samples were measured using high performance liquid chromatography (HPLC) (Waters 1525, Waters, United States) equipped with a refractive index detector (Waters 2414, Waters, United States) with an Aminex HPX-87H column (300 × 7.8 mm) and a Cation H Cartridge Micro-Guard column (Bio-Rad, USA), using the method as described by Li et al. [22]. The column temperature was set as 60 °C and 5 mM H_2_SO_4_ was used as the mobile phase at a flow rate of 0.6 mL/min.

Furfural, 5-hydroxymethylfurfural (5-HMF), vanillin, syringaldehyde, *p*-coumaric acid and ferulic acid were analyzed and quantified by HPLC using a Aminex HPX-87H column (300 × 7.8 mm) and a Cation H Cartridge Micro-Guard column (Bio-Rad, United States) at 60 °C equipped with a UV-detector (Waters 2487, Waters, United States). As mobile phase, 5 mM H_2_SO_4_ at a flow rate of 0.6 mL/min was used.

The total phenolics were measured using a Folin-Ciocalteu method [23]. Phloroglucinol dehydrate was used as a standard. For detail operations, 20 µL of diluted sample was mixed with 100 µL of Folin-Ciocalteu reagent (Sangon Biotech, China) and incubated at room temperature for 5 min in dark conditions. Then 80 µL of 7.5% Na_2_CO_3_ was added and mixed. After 2 h incubation at room temperature in the dark, the absorbance was measured at 750 nm with a EnSpire-2300 multimode plate reader (PerkinElmer, USA).

The data were analyzed statistically by one-way analysis of variance (ANOVA) with Duncan’s multiple-range test. SPSS for windows (SPSS Inc. Chicago, version 17.0) was used for all statistical analysis and a value of *P* < 0.01 was considered significant.

## Results and discussion

### Isolation and identification of the hydrogen production strain MJ1

An enrichment culture was established by incubating paper sludge in MB medium (xylose as the carbon source) at 55 °C. One bacterial strain called MJ1 was isolated for its fast growth and the ability to produce hydrogen in xylose medium. The paper sludge was the residue of papermaking raw materials from which hemicellulose and lignin were removed with a kraft process. Undoubtedly, the xylan was present in the paper sludge and it is an ideal source to screen the xylose or xylan-utilizing strains. As some inhibitors may also exist in paper sludge, there is an opportunity to screen out inhibitor-tolerant strains.

**Fig. 1:**
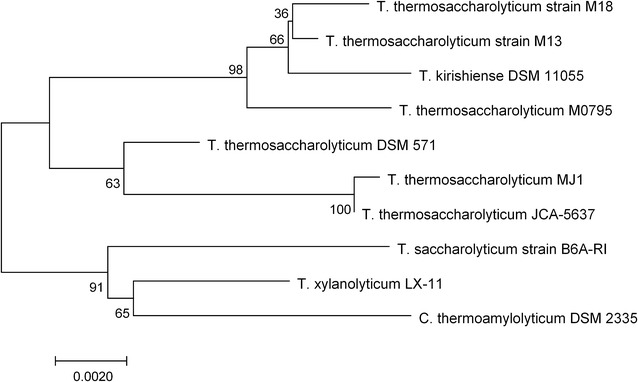
Phylogenetic relationship of strain MJ1 and other *Thermoanaerobacterium* strains based on 16S rRNA gene sequences. *Numbers* along branches indicate bootstrap values with 1000 times

An analysis of the 16S rRNA gene sequence of MJ1 indicated that the strain is a member of the genus *Thermoanaerobacterium*. The closest phylogenetic relative was *T. thermosaccharolyticum* JCA-5637, with 99% similarity to the 16S rRNA genes. A phylogenetic tree was constructed (Fig. 1). Similar to other strains of the genus *thermosaccharolyticum*, MJ1 cells are oval shaped (Additional file 1: Fig. S1), tufted flagellum, motile, and exhibit anaerobic growth.

### Effect of different sugars on growth, hydrogen production and metabolite production via strain MJ1

The strain *T. thermosaccharolyticum* is a thermophilic saccharolytic microorganism that could utilize various types of carbohydrate as carbon source. Effective fermentative bacteria should be able to use various types of substrates, especially complex carbohydrate. Therefore, four common sugars (glucose, cellobiose, xylose and sucrose) were chosen to test the utilization ability of MJ1. The growth curves are shown in Additional file 2: Fig. S2. The MJ1 can utilize glucose, cellobiose and xylose except sucrose. This was different from previous studies reporting that sucrose can be utilized by other *T. thermosaccharolyticum* stains [8, 10, 12, 19, 24]. This may be another evidence to demonstrate that MJ1 is a novel strain within the species *T. thermosaccharolyticum*. When utilizing xylose as the carbon source, the OD_600_ value was lower and gradually reached those of glucose and cellobiose. Although the final OD_600_ value of xylose was still lower, the performance gap was small, indicating that MJ1 could grow well in xylose medium. MJ1 could not utilize Avicel to maintain the growth (data not shown).

**Fig. 2:**
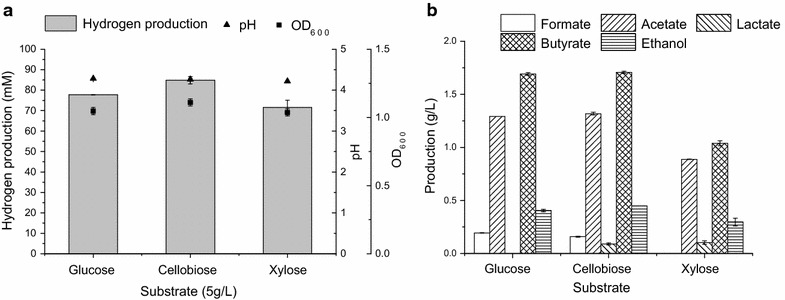
Hydrogen production and metabolite production via strain MJ1 with different sugars. **a** Hydrogen production, final pH and OD_600_ of MJ1 with three types of sugars. **b** Metabolite production of MJ1 with three types of sugars

The hydrogen production level of MJ1 from glucose, cellobiose and xylose was examined. The theoretical maximum hydrogen yields were 4 mol-H_2_/mol-glucose and 3.33 mol-H_2_/mol-xylose in acetate type fermentation [25, 26]. With the concentration of each substrate fixed at 5 g/L, MJ1 could efficiently transform those sugars to hydrogen. After the fermentation, 66% of glucose, 52% of cellobiose and 98% of xylose were consumed by MJ1, respectively. The hydrogen production is shown in Fig. 2a. The hydrogen yield was calculated excluding residual sugars in the broth (glucose about 1.7 g/L, cellobiose about 2.4 g/L and xylose about 0.1 g/L). The maximum total hydrogen production and hydrogen yield were reached from cellobiose (1902 ± 40 mL-H_2_/L, 731 ± 15 mL-H_2_/g-cellobiose and 11.18 ± 0.25 mol-H_2_/mol-cellobiose), followed by glucose (1741 ± 4 mL-H_2_/L, 528 ± 1 mL-H_2_/g-glucose and 4.25 ± 0.01 mol-H_2_/mol-glucose) and xylose (1603 ± 79 mL-H_2_/L, 321 ± 15 mL-H_2_/g-xylose and 2.15 ± 0.11 mol-H_2_/mol-xylose). In this study, 2 g/L yeast extract and 2 g/L peptone were added in fermentation medium and they might contribute to a higher hydrogen yield. *Thermoanaerobacterium* spp. has been demonstrated to have the ability to consume complex substrates to produce hydrogen, such as cellulose [10, 11], starch [5, 19], xylan [5], glucose [8, 19, 24], xylose [8, 19, 27] and sucrose [8, 13, 28]. The corresponding hydrogen yields from different isolated *T. thermosaccharolyticum* stains are listed in Table 1. The hydrogen yield of MJ1 was comparable to that of other strains when using xylose as the substrate [8, 19, 27]. With glucose used as the substrate, the hydrogen yield of MJ1was much higher than that of W16, PSU-2 and TERI S7 [8, 19, 28], proving that glucose can be well utilized by MJ1 and is suitable for hydrogen production. Li et al. [2] and Ren et al. [7] have proposed strategies for hydrogen production from lignocellulose by *T. thermosaccharolyticum* which lacks the ability to degrade lignocellulose, implying that MJ1 is a good candidate for hydrogen production.

**Table 1:** Comparison of hydrogen yield in sugar fermentation by *T. thermosaccharolyticum*

Substrate	Strain	Hydrogen yield (mol H_2_/mol substrate_consumed_)	Reference
Glucose	W16	2.42 ± 0.11	[19]
	PSU-2	2.43 ± 0.04	[28]
	TERI S7	2.5 ± 0.2	[8]
	MJ1	4.25 ± 0.01	Present study
Cellobiose	MJ1	11.18 ± 0.25	Present study
Xylose	KKU19	2.09 ± 0.02	[27]
	W16	2.19 ± 0.12	[19]
	TERI S7	2.2 ± 0.2	[8]
	MJ1	2.15 ± 0.11	Present study

The metabolites in the broth were formate, acetate, lactate, butyrate and ethanol (Fig. 2b). Formate was absent in xylose medium and lactate was absent in glucose medium. The total metabolite production was much lower in xylose medium than in glucose or cellobiose medium. Acetate and butyrate were the major metabolites, accounting for more than 80% of the production. Butyrate produced in the fermentation could enhance the hydrogen yield, and the ratio of butyrate to acetate concentration (B/A) is frequently used as an indicator to evaluate the effectiveness of hydrogen production [29, 30]. Ren et al. [19], Khamtib et al. [27] and Singh et al. [8] have also reported that acetate, butyrate and ethanol were the predominant metabolites. However, Singh et al. failed to observe formate and lactate during fermentation, probably due to the variation in the performance of different strains and substrates [8]. The results of metabolites indicated that the strain MJ1 produces hydrogen mainly through the acetate and butyrate pathway.

### Effects of xylose concentrations on hydrogen production and metabolite production via strain MJ1

**Fig. 3:**
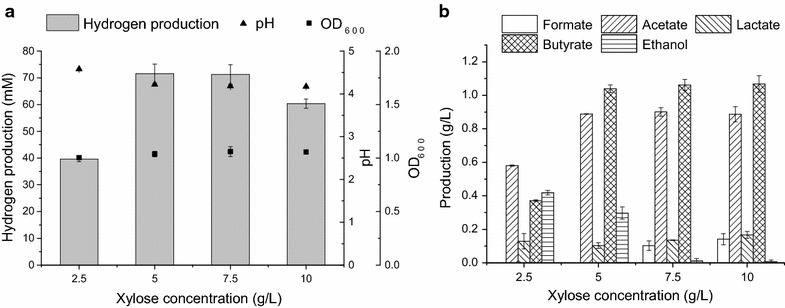
Hydrogen production and metabolite production via strain MJ1 with different xylose concentrations. **a** Hydrogen production, final pH and OD_600_ of MJ1 with different xylose concentrations. **b** Metabolite production of MJ1 with different xylose concentrations

As shown in Table 1, MJ1 could efficiently utilize xylose to produce hydrogen, and the effect of xylose concentration on hydrogen production was investigated. Generally, substrate concentration largely affects the hydrogen production [10, 27]. The initial xylose concentration affected the production of hydrogen and metabolites (Fig. 3). The hydrogen production was significantly increased from 2.5 to 5 g/L, followed by a slight decrease at 10 g/L, probably due to substrate and product inhibition [19, 27, 28]. Under the test conditions, the maximum hydrogen yield (2.38 ± 0.06 mol-H_2_/mol-xylose) and hydrogen production (71.57 ± 3.55 mM) occurred at 2.5 and 5 g/L, respectively. The hydrogen yield at 5 g/L (2.15 ± 0.11 mol-H_2_/mol-xylose) was comparable to that at 2.5 g/L and the hydrogen production was significantly increased from 2.5 to 5 g/L (39.64 ± 0.95 to 71.57 ± 3.55 mM). An increase in substrate concentration could increase hydrogen production up to a certain level due to the production of acetic and butyric acids [27]. At a concentration exceeding 5 g/L, the xylose would remain in the broth, indicating that 5 g/L of xylose was economical for hydrogen production. The production of metabolites was also significantly increased from 2.5 to 5 g/L and showed no obvious differences at higher xylose concentrations. The metabolite production maintained relative consistency at higher xylose concentrations possibly due to the pH change that inhibited the metabolism of MJ1. The production of acetic and butyric acids is correlated with hydrogen, and the metabolite production demonstrated that 5 g/L of xylose was suitable for hydrogen production at the same time.

### Optimization of culture conditions for hydrogen production from DAPSBH

The DAPSBH was obtained from the dilute-acid pretreatment process of lignocellulose. Due to low pH and high inhibitor concentration (usually 0.79–4.06 g/L acetate, 0.05–2.94 g/L furfural and 0.01–0.53 g/L 5-HMF) [31–35], DAPSBH is usually discarded by researchers. Hemicellulose can be effectively disrupted during the acid pretreatment process, and high concentration of xylose (usually 1.2–19.1 g/L) exists in DAPSBH. The discard of DAPSBH will not only cause the waste of resources but also pollute the environment. Cao et al. have investigated the hydrogen production from the cornstalk acid hydrolysate with detoxification by Ca(OH)_2_ [13], and up to now, some studies have been carried out for hydrogen production from non-detoxified hydrolysate [15–18]. Furthermore, the inocula were all mixed cultures from sludges, enriched hydrogenogenic culture or methonogenic granules, indicating that the inhibitor-tolerant strains did exist in natural environment.

**Fig. 4:**
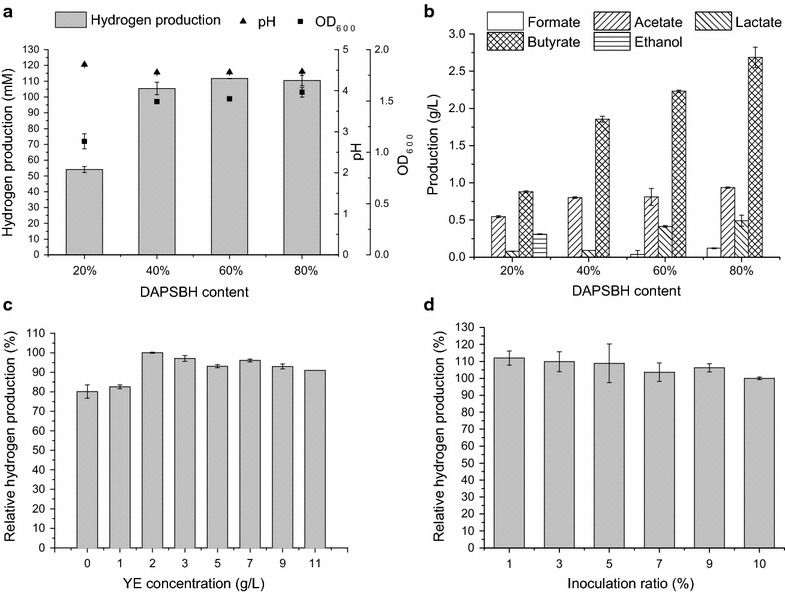
Hydrogen production and metabolites production via strain MJ1 with DAPSBH. **a** Hydrogen production, final pH and OD_600_ of MJ1 with different DAPSBH concentrations. **b** Metabolite production of MJ1 with different DAPSBH concentrations. **c** Hydrogen production with different YE concentrations. The hydrogen production with 2 g/L YE addition was used as the control and defined 100%. **d** Hydrogen production with different inoculation ratios. The hydrogen production under inoculation ratio of 10% was used as the control and defined 100%

The DAPSBH used in this study consisted of 1.54 ± 0.58 g/L glucose, 12.03 ± 0.35 g/L xylose and 2.53 ± 0.09 g/L acetate. As for inhibitors, there were 123.50 ± 3.54 mg/L furfural, 19.50 ± 3.53 mg/L 5-HMF, 4.50 ± 0.71 mg/L vanilline, 8.00 ± 2.83 mg/L syringaldehyde, 65.50 ± 2.12 mg/L *p*-coumaric acid, 67.00 ± 2.83 mg/L ferulic acid and 725.49 ± 20.79 mg/L total phenolics in DAPSBH. According to the results of xylose concentrations for the hydrogen production, the DAPSBH was diluted and supplied nutrients of MB medium. The hydrogen production is shown in Fig. 4a and exhibited a similar trend to that of different xylose concentrations. The hydrogen production showed no significant increase (P = 0.23 > 0.01) among 40, 60 and 80% of DAPSBH and was significantly increased (P = 0.003 < 0.01) from 20 to 40% of DAPSBH, probably due to the increase of substrates and the cell concentration (with OD_600_ increased from 1.11 to 1.49). The hydrogen production was higher in DAPSBH than that at a similar xylose concentration. For example, the hydrogen production from 40% of DAPSBH (equivalent to 5 g/L xylose) reached 105.42 mM, which was much higher than 71.57 mM from 5 g/L xylose. The higher hydrogen production from DAPSBH suggests that the inhibitors do not influence the growth of MJ1 and DAPSBH is more suitable for hydrogen production using MJ1 than single xylose, even glucose and cellobiose. DAPSBH also showed higher production of metabolites. The enhanced butyrate production means higher butyrate/acetate ratio, which is beneficial to hydrogen production [29, 30]. This will also increase hydrogen production on a same sugar-equivalent basis. The higher cell concentration and butyrate production may be the primary reasons for the high hydrogen production.

As addition of yeast extract (YE) will increase the cost of hydrogen production from lignocellulose and hydrogen production would be inhibited by high nitrogen source concentration [36, 37], the concentration of YE was optimized for hydrogen production. As shown in Fig. 4c, hydrogen production was improved with the YE concentration increased from 0 to 2 g/L, but with its concentration beyond 2 g/L, the hydrogen production gradually decreased. The maximum hydrogen production was obtained at 2 g/L (118.92 ± 0.24 mM).

The inoculation ratios will influence the cost of seed solution and hydrogen production as well. As shown in Fig. 4d, hydrogen production had no significant differences among different inoculation ratios (P = 0.55 > 0.01) and even obtained a higher hydrogen production at low inoculation ratios. This is a good sign for scale-up fermentation, which will considerably decrease the workload for the seed solution preparation. This phenomenon may be attributed to the reason that lower metabolites in seed solution were transferred to the fermentation broth, leading to the fast growth of MJ1 in DAPSBH.

### Time course of hydrogen production and metabolite production via strain MJ1 at 60% DAPSBH

**Fig. 5:**
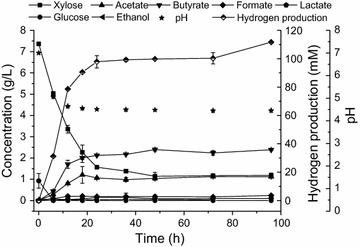
Time course of hydrogen production and metabolite production via strain MJ1 at 60% DAPSBH

Hydrogen production, metabolite production and substrate consumption from 60% DAPSBH were investigated. As shown in Fig. 5, when MJ1 was incubated in the 60% DAPSBH, hydrogen was quickly produced after inoculation. The relative maximum hydrogen production was obtained at 24 h, followed by a slight increase, and the highest hydrogen production was obtained at 96 h. The hydrogen production rate reached 6.55, 5.03 and 4.08 mmol-H_2_/L h at 12, 18 and 24 h, respectively. The hydrogen production rates were comparable with those of other studies using soluble sugar as carbon source [19, 27]. The concentration of sugars gradually decreased with hydrogen accumulation. Glucose was exhausted in the initial 6 h and 30% xylose was consumed at the same time, indicating that MJ1 might utilize glucose and xylose simultaneously or directly utilize xylose when glucose was used up. The predominant metabolites were acetate and butyrate besides hydrogen, suggesting that the MJ1 followed the acetate butyrate pathway for hydrogen production, which was consistent with previous studies [8, 27, 28]. As shown in Fig. 5, the hydrogen was simultaneously produced with acetate and butyrate. With the increase of incubation time, the amount of accumulated acetate and butyrate also increased with increasing hydrogen production. The pH value was dropped rapidly in the first 12 h and then remained at a relative stable level.

### Comparison of hydrogen production from sugars and DAPSBH

**Fig. 6:**
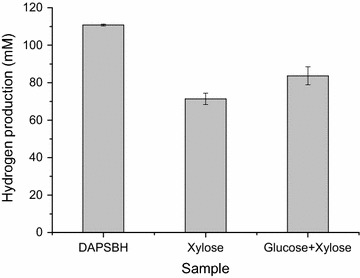
Comparison of hydrogen production from sugars and DAPSBH

DAPSBH showed significantly higher hydrogen production than the equivalent amount of xylose. Apart from xylose, there was about 1 g/L glucose in 60% DAPSBH. The contribution of existing glucose on the hydrogen production was investigated. In order to mimic the sugar component of DAPSBH, a mixture of glucose and xylose was used. As shown in Fig. 6, the combination of glucose and xylose can enhance hydrogen production. Although the hydrogen production of mixed sugars reached 86.68 ± 3.03 mM with an increase of 17.22% over that of xylose, it was only 75.72% that of DAPSBH. The hydrogen production of mixed sugars demonstrated that the existing glucose in DAPSBH was not the primary reason for the higher hydrogen production of DAPSBH.

### Effects of pH and CaCO_3_ buffer system on hydrogen production via strain MJ1

**Fig. 7:**
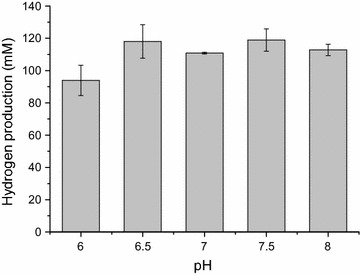
Effects of pH on hydrogen production via strain MJ1

As pH plays a very significant role in determining the type of fermentation pathway in anaerobic biohydrogen processes [38], the effect of initial pH on hydrogen production from DAPSBH was investigated within the pH range of 6.0–8.0 with an increment of 0.5. As shown in Fig. 7, hydrogen production was related to initial pH value and did not show a general trend. The higher hydrogen production was obtained at 6.5 and 7.5 (118.04 ± 10.38 and 118.92 ± 6.94 mM). There was no significant difference in hydrogen production within the initial pH range of 6.5–8.0 and a relative high hydrogen production was obtained at initial pH 6.0. Too high or too low pH inhibited the activity of FeFe-hydrogenase, resulting in low hydrogen production [39]. This agreed partially with several previous studies reporting that the initial pH ranges of 5.5–6.5, 7.0–8.0 or 6.0–7.0 were the optimal for hydrogen production by *T. thermosaccharolyticum*, and hydrogen production was rapidly falling with the initial pH out of the optimum range [10, 19, 27]. This implied that MJ1 had an excellent pH range for industrialization of biohydrogen production.

**Fig. 8:**
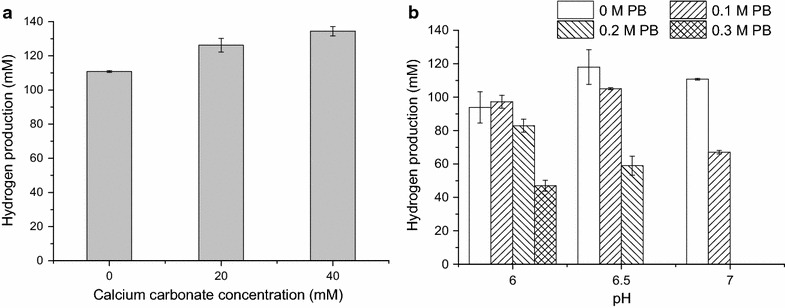
Effects of CaCO_3_ and buffer system on hydrogen production via strain MJ1. **a** Effects of CaCO_3_ on hydrogen production via strain MJ1. **b** Effects of buffer system on hydrogen production via strain MJ1

CaCO_3_ played a very important role in enhancing the production of hydrogen. The calcium supplementation can be an effective way to improve the hydrogen production under anaerobic conditions by *Clostridium pasteurianum* or seed sludge, and the hydrogen production was similar for different forms of Ca^2+^ [40, 41]. CaCO_3_ supplementation resulted in high solvent formation and hydrogen production by stimulating the electron transport system mediated by protein bound FAD, 4Fe–4S, NADH and flavoproteins. The other reason for stimulatory effect of CaCO_3_ on biohydrogen production was mainly attributed to the buffering capacity of carbonate [42]. The CaCO_3_ can maintain the medium at a relative higher pH. The stimulatory effect of CaCO_3_ on hydrogen by MJ1 was investigated. As shown in Fig. 8a, the hydrogen production was significantly enhanced by CaCO_3_ (P = 0.008 < 0.01). At CaCO_3_ concentration of 40 mM, the hydrogen production reached 134.42 ± 2.72 mM (3.11 L-H_2_/L), a 21.32% increase over the control. The final pH of the broth was 5.03 for 20 mM CaCO_3_ and 5.31 for 40 mM CaCO_3_. Those indicated that the pH of broth can be buffered by CaCO_3_. The CaCO_3_ also showed a positive effect on hydrogen production by MJ1. Apart from Ca^2+^ ions, the buffering capacity of CaCO_3_ (higher final pH) also played an important role in hydrogen production.

In order to gain insight into higher hydrogen production like supplementation of CaCO_3_, we investigated the effect of buffer system on the hydrogen production via MJ1 using the low cost PB. As shown in Fig. 8b, the PB significantly influenced the hydrogen production, especially at higher PB concentrations. With the addition of 0.1 M PB, the hydrogen production was not significantly influenced at pH 6.0 and 6.5, but obviously decreased at pH 7.0. Hydrogen could not be detected with the addition of 0.2 M PB (pH 7.0) and 0.3 M PB (pH 6.5 and 7.0), because MJ1 could not grow under those conditions (the media remained clear all the time). The high PB concentration (higher salt ionic strength) inhibits the growth and hydrogen production of MJ1. With PB existing in media, MJ1 had a better hydrogen production in acidic environment than in alkalic environment. The results indicated the PB system was not suitable for the hydrogen production and the pH control in the bioreactor would be an ideal way to maintain the pH for higher hydrogen production.

## Conclusions

*Thermoanaerobacterium thermosaccharolyticum* MJ1, a strictly anaerobic thermophilic bacterium, was isolated from paper sludge. The lag phase of hydrogen production was not observed for MJ1 when a mixture of glucose and xylose was used as carbon source. More important, MJ1 had an excellent tolerance to inhibitors and could directly utilize DAPSBH from 1% dilute-acid pretreatment without detoxification for hydrogen production, with a yield higher than that of pure sugars. The higher butyrate/acetate ratio and cell concentration may be the primary reasons for high hydrogen production in DAPSBH. Supplementation of CaCO_3_ had a stimulatory effect on the hydrogen production by MJ1, and PB system was not suitable for pH control in the hydrogen production. The present study indicates that MJ1 might be an excellent candidate for economic biohydrogen production from non-detoxified DAPSBH. This study also offers a new idea for isolating novel strains that could overcome the problems existing in the fields of biofuels.

## Additional files

**Additional file 1: Figure S1.** Atomic force microscope (AFM) image of *T. thermosaccharolyticum* MJ1.

**Additional file 2: Figure S2.** Growth curve of MJ1 with different sugars.

## Abbreviations

SCB: sugarcane bagasse
DAPSBH: dilute-acid pretreated sugarcane bagasse hydrolysate
PB: phosphate buffer
5-HMF: 5-hydroxymethylfurfural
