# Supplementary figures and images for: Direct hydrogen production from dilute-acid pretreated sugarcane bagasse hydrolysate using the newly isolated Thermoanaerobacterium thermosaccharolyticum MJ1
Source: Microb Cell Fact. 2017 May 3;16:77. doi: 10.1186/s12934-017-0692-y (PMC5415828; doi:10.1186/s12934-017-0692-y)

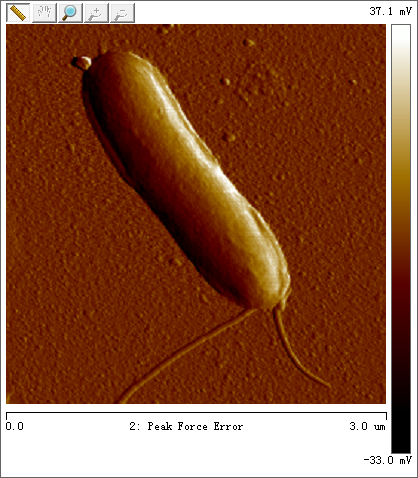

Supplement: Supplementary file 1 — Additional file 1: Figure S1. Atomic force microscope (AFM) image of T. thermosaccharolyticum MJ1. [file 12934_2017_692_MOESM1_ESM.docx]
