# Supplementary material for: Direct hydrogen production from dilute-acid pretreated sugarcane bagasse hydrolysate using the newly isolated Thermoanaerobacterium thermosaccharolyticum MJ1
Source: Microb Cell Fact. 2017 May 3;16:77. doi: 10.1186/s12934-017-0692-y (PMC5415828; doi:10.1186/s12934-017-0692-y)
Supplement: Supplementary file 2 [file 12934_2017_692_MOESM2_ESM.docx]

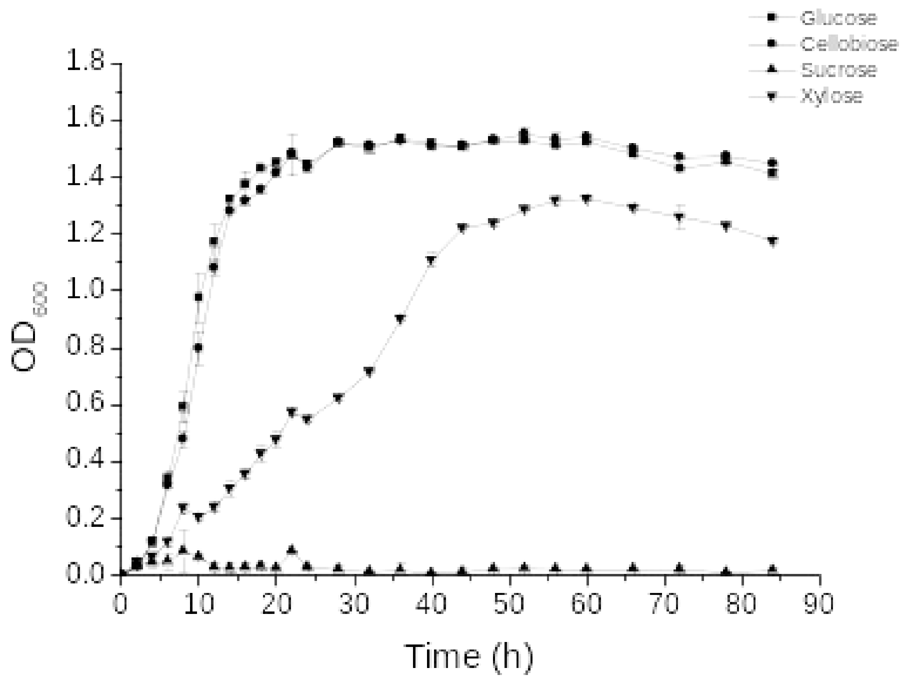
Glucose
Cellobiose
Sucrose
Xylose
OD600
Time (h)
1.8
1.6
1.4
1.2
1.0
0.8
0.6
0.4
0.2
0.0
0
10
20
30
40
50
60
70
80
90
